# Editorial: Role of the serotonergic system in pathology of major depressive disorders

**DOI:** 10.3389/fpsyt.2022.988307

**Published:** 2022-08-08

**Authors:** Trevor Ronald Norman

**Affiliations:** Department of Psychiatry, Austin Hospital, University of Melbourne, Heidelberg, VIC, Australia

**Keywords:** depression, inflammation, genetics, MAO, 5HT receptors, serotonin

Serotonin neurons, originating in the raphe nuclei and innervating multiple brain regions, have been implicated in diverse behavioral and cognitive processes. Moreover, evidence suggests that during the development of the central nervous system serotonin, acting through its 14 different receptors, influences synaptic organization (1). This ability of the system to reorganize and rewire seems to persist into adulthood, where following insults, such as chronic stress, the serotonergic system undergoes profound changes. This is particularly evident in the development of major depressive disorder where there is evidence for the rewiring of the system (Albert and Vahid-Ansari). The amplification of serotonin activity during treatment with antidepressant medications may be critical to the plasticity of the system which in turn, leads to improvements in mood and cognitive symptoms. Animal studies have shown that endogenous serotonergic tone can markedly influence behavioral outcomes in rodents following treatment with fluoxetine a predominantly serotonergic agent (Kesic et al.). Together two these papers reinforce not just the significance of serotonin in depressive disorders and its influence on behavioral outcomes, but the importance of potential differences in effects in regional brain areas. Furthermore, this later study showed an upregulation of 5HT4 receptors and a concomitant down regulation of 5HT1A receptors in prefrontal cortex following chronic fluoxetine administration. The result with the 5HT4 receptor resonates with the findings in healthy volunteers of the sub-chronic administration of prucalopride, a 5HT4 partial agonist, on cognitive function (De Cates et al.). Using functional MRI subjects receiving prucalopride were more accurate at identifying the gender of faces in a rapidly presented faces task. This finding was consistent with a pro-cognitive effect of the drug. The improvement in behavioral response was accompanied by reduced activation of the default mode network. This network is reported to be activated in patients with depression. While these results do not of themselves indicate an antidepressant effect of the 5HT4 partial agonist, coupled with the results from pre-clinical studies (2), they suggest that further investigations of this class of agent may be warranted. In particular the use of the drug may be of benefit to the alleviation of cognitive symptoms occurring in depression.

The hypothesis, now decades old, that serotonin participated in the etiology of major depression (and other mental disorders) led to the progress in treatments for the disorder based on restoration of central serotonin function. Development of the Selective Serotonin Reuptake Inhibitors or SSRI group of antidepressant medications grew from this realization. In recent times this class of agent has become the most widely prescribed treatment of depressive episodes and first line treatment for most anxiety disorders. Further investigation of the role that the multiplicity of serotonin receptors play in the function of the central nervous system has resulted in the development of a more nuanced view of the neurobiological basis of anxiety and depressive disorders. More recently, alterations in events occurring beyond the receptor have noted the importance of neurotrophic factors and the alteration of gene expression as a significant explanatory mechanisms of both antidepressant actions and of depression itself (3).

Alternative postulates of depression etiology include the so called “cytokine hypothesis” in which the increase of pro-inflammatory cytokines in the central nervous system can activate microglial cells leading to oxidative stress and neuronal damage. Taken at face value such a hypothesis appears remote from any involvement of serotonin. On the other hand, inflammatory molecules activate the enzyme indole diamine oxidase (IDO) which in turn depletes central serotonin concentrations by shunting serotonin into an alternative metabolic pathway. The end products of the alternate pathway are kynurenine and the glutamate agonist, quinolinic acid. Antidepressant medications oppose this pathway and restore serotonin function. While the role of inflammation in major depression is far from settled accumulating evidence suggests that several inflammatory cytokines in particular TNF-α, IL-6 and C-reactive protein are elevated in patients with major depression (4). Zhu et al. report evidence for decreased serum concentrations of interlukin-8 (IL-8) in patients with major depressive disorder compared to controls. This difference was more pronounced for drug free patients, whereas those patients receiving treatment with SSRI medications were not different to healthy controls. This later finding may point to the “normalization” of cytokines with effective pharmacotherapy. Interestingly concentrations of IL-8 were negatively correlated with depression scores for the whole group of patients. The data add to the accumulating body of evidence for the role of IL-8 in major depression. A recent review notes the contradictory literature for individual studies of this cytokine in depression (5), but a meta-analysis suggested an overall decrement of IL-8 in depression (6). This begs the question of the mechanism by which the pro-inflammatory cytokine is decreased in the face of an inflammatory hypothesis of depression etiology. Perhaps the notion that IL-8 (among other cytokines) can act as a chemoattract agent for neural precursor cells under specific conditions might suggest that low levels would impede neurogenesis (7). Such an explanation would be entirely consistent with the neurotrophic hypothesis of depression (8).

The role of serotonin in suicide and suicide attempts has been investigated since the pioneering studies indicating lowered concentration of the serotonin metabolite, 5-hydroxyindole acetic acid (5HIAA), in the cerebrospinal fluid (CSF) was a potential risk factor for completed suicide in depressed patients (9). More recent studies have focussed attention on the association of serotonergic genetic markers and suicide. Thus, Yang et al., have performed a meta-analysis of polymorphisms of the 5HT1B gene and their association with risk of depression, response to medication and suicidal behavior. Subjecting the 21 relevant studies to the meta-analysis the authors concluded that a significantly increased risk of depression was associated with the rs6296 GC and GC/CC genotypes while the rs6298 CT genotype was significantly associated with an increased risk of suicidal behavior. The 5HT1B receptor functions as a presynaptic auto-receptor and a post-synaptic heteroreceptor which influences a number of neurotransmitters systems involved in psychopathology (10). Although the exact role of these receptors in depression is not certain it is of note that antidepressants reduce 5HT1B mRNA in the dorsal raphe leading to increased release of 5HT, while stress upregulates 5HT1B receptors (11). Furthermore, preclinical studies have demonstrated antidepressant effects of 5HT1B agonists (10). Direct measurement of serotonin concentrations in peripheral fluids such as saliva, plasma, serum or platelets and the attempt to relate these to the presence of depression has yielded conflicting data (12, 13). Given that most of peripheral serotonin is generated in the gut it has been difficult to establish a direct relationship to concentrations in the central nervous system. Nevertheless, peripheral fluids, saliva in particular, are a less invasive means of sampling serotonin directly. The demonstration of an inverse correlation between mood and salivary serotonin in a group of healthy young athletes suggests further development may be of interest in monitoring depressive symptoms (Karbownik and Hicks).

The direct relationship between serotonin metabolism and the activity of the enzyme monoamine oxidase-A (MAO-A) is well recognized. Moreover, MAO-A activity is genetically determined and various polymorphisms have been associated with antisocial behavior and aggression (14). In a multi-center study of more than 800 patients with major depressive disorder Ludwig et al. examined the association of polymorphisms of the MAO-A gene, methylation status of Exon 1 of the gene and violent suicide attempts. Dysregulation of the MAO-A gene was suggested at both a genetic and epigenetic level in female patients with a personal history of violent suicide attempt(s). The decreased methylation status in this sample may be associated with increased expression of MAO-A in the brain resulting in decreased availability of serotonin.

## Author contributions

The author confirms being the sole contributor of this work and has approved it for publication.

## Conflict of interest

The author declares that the research was conducted in the absence of any commercial or financial relationships that could be construed as a potential conflict of interest.

## Publisher's note

All claims expressed in this article are solely those of the authors and do not necessarily represent those of their affiliated organizations, or those of the publisher, the editors and the reviewers. Any product that may be evaluated in this article, or claim that may be made by its manufacturer, is not guaranteed or endorsed by the publisher.
